# New method of measuring wrist joint position sense avoiding cutaneous and visual inputs

**DOI:** 10.1186/1743-0003-7-5

**Published:** 2010-02-10

**Authors:** Andre Gay, Kimberly Harbst, Kenton R Kaufman, Diana K Hansen, Edward R Laskowski, Richard A Berger

**Affiliations:** 1 Biomechanics Laboratory, Division of Orthopedic Research, Mayo Clinic, 200 First Street SW. Rochester, MN 55095, USA

## Abstract

**Background:**

Aspects of afferent inputs, generally termed proprioception, are being increasingly studied. Extraneous factors such as cutaneous inputs can dramatically interfere while trying to design studies in order to determine the participation of the different structures involved in proprioception in the wrist position sense. We tried to determine validity and repeatability of a new wrist joint position measurement device using methodology designed to minimize extraneous factors and isolate muscle and joint inputs.

**Methods:**

In order to test the reliability of the system, eighty young-adult subjects without musculoskeletal or neurologic impairments affecting the right upper extremity were tested using a custom made motion tracking system. Testing consisted of two conditions: active reproduction of active placement and passive reproduction of passive placement. Subjects performed two repetitions of each target position (10, 20, and 30° of flexion and extension) presented in a random order. Test- retest reliability was then tested.

**Results:**

The average constant error in the passive condition was -0.7° ± 4.7° as compared to the active condition at 3.7° ± 5.1°. Average absolute error in the passive condition was 4.9° ± 2.9° compared to the active condition in which absolute error was 5.9° ± 3.5°.

**Discussion:**

Test-retest repeatability in both conditions was less than the 5° magnitude typical of clinical goniometry. Errors in the active condition (less than 2°) were slightly smaller than the passive condition, and the passive condition was also associated with poorer consistency between apparatus sensors and skin sensors.

**Conclusions:**

The current system for measurement of wrist joint proprioception allows the researcher to decrease extraneous influences that may affect joint position sense awareness, and will help in future study aiming to determine precisely the role of the different structure involved in proprioception.

## Background

Aspects of afferent inputs, generally termed proprioception, are being increasingly studied in an attempt to describe and understand impairments [1], to optimize rehabilitation effectiveness following trauma or surgery [1], and to prevent recurrent injury [2-9]. Results of previous studies have led to the conclusion that proprioception is multi-faceted and that multiple sensory receptors generate afferent proprioceptive inputs: Visual [10-14], muscle spindle [15-17], cutaneous [18], tendon and joint [19]. All these receptors have each been demonstrated to contribute to the sense of position or motion of a body part in space [20,21]. Isolating each proprioceptive input from specific structures in order to determine the effect of disease or injury has proven to be difficult. Clarification of the role and importance of a specific structure such as muscle spindles afferents is essential to understanding the potential impact of surgeries or injuries that diminish or destroy those structures [8,22,23].

Methodology differs greatly between studies and even within studies in which a body part is positioned as a target and the same or contralateral body part is positioned to match. Studies also vary in their means of setting the target position, and active, passive, or active-assisted motion may be employed. Regardless of the method used to achieve the target position, the target reproduction may be accomplished by active or passive methods. Theoretically, pairing different types of motions [24-27] could result in a confounding effect. None of these methods of assessing target position reproduction has been adopted as a standard, which likely contributes to variability of results.

Reliability measures for proprioception testing have been in the .85-.95 range at more proximal joints [27-31]. Redundant sensory information may, however, allow the subject to produce more reliable results than may be afforded if extraneous factors are minimized. Techniques utilized to measure joint angles or limb position also present potential confounding factors.

In previous studies, researchers have objectively documented joint position sense using dynamometers [32,33] electrogoniometers, potentiometers [34], electromagnetic sensors [6,21,35], and video digitization/analysis [36]. Reproducibility of wrist motion measurement using a simple goniometer was reported as 5-8° (intra-observer) and 6-10° (inter-observer) [37]. At the elbow joint, reliability using the electrogoniometer was shown to be superior to either a universal goniometer or a fluid goniometer [38]. The repeatability of electromagnetic sensors is anticipated to be superior to standard goniometric measurements, but has not been demonstrated for the wrist.

The purpose of this study is to formulate a valid and repeatable method for testing wrist joint position sense avoiding stimulating cutaneous inputs. Optimal methodology entails isolating muscle spindles and/or joint receptors contribution to proprioception at the wrist while minimizing extraneous influences using a non-invasive method.

## Methods

**Figure 1:**
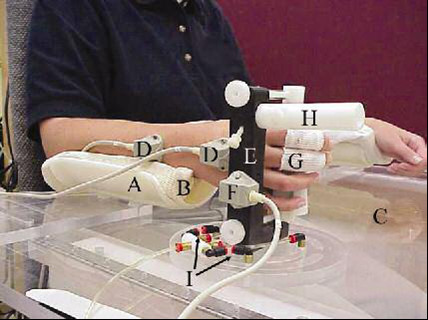
**Subject seated in experimental apparatus**. Components of the apparatus are labeled: A) Forearm support, B) Slow-recovery foam, C) Plexiglass desktop, D) Skin Sensors, E) Manipulandum, F) Manipulandum Sensors, G) Finger tubular dressings, H) Manipulandum projection, I) Air jets.

**Figure 2:**
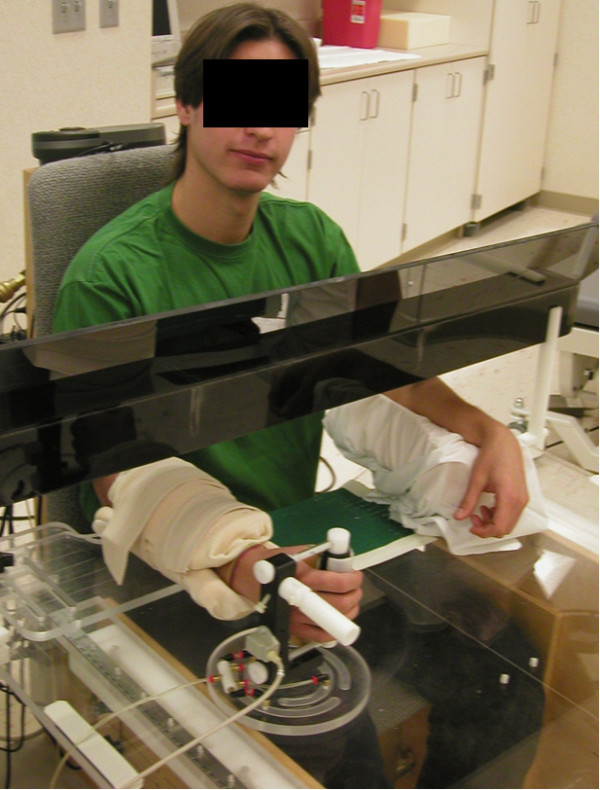
**Close up of experimental apparatus with foam forearm stabilization and straps in place**.

Subjects participating in this study included eighty, healthy, 20-65 year-old volunteers. This study was approved by our Institutional Review Board. Informed consent to participate was obtained. Each subject was seated in a custom-made chair with both forearms supported on armrests attached to a plexiglass desktop (Figure 1). The forearm supports were lined with 3 cm thick, slow-recovery, viscoelastic foam. The subject's right arm was positioned in neutral forearm pronation/supination and neutral wrist flexion/extension. Thus, wrist motion occurred in a plane parallel to the desktop. Two electromagnetic sensors (Flock of Birds; Ascension Technology, Burlington, VT) were attached to the dorsal forearm and the middle of the dorsal aspect of the third metacarpal to measure wrist flexion/extension. An additional 1.5-inch thick piece of foam was placed over the length of the right forearm and two straps encircled the forearm and foam to secure the forearm to the armrest (Figure 2) and isolate wrist motion while decreasing extraneous cutaneous input. Finger lengths of tubular dressings (Xspan^®^) with hook surfaced Velcro^® ^strips attached were applied to the right thumb, index, and middle fingers. The subject was asked to gently grip a vertical upright cylindrical experimental apparatus (manipulandum) covered in loop-surfaced Velcro^® ^with a 3/4-inch dowel rod interposed within the first web space to minimize wrist extension with finger flexion due to tenodesis. The subject was instructed to relax and the wrist joint position was measured with a plastic goniometer to assure neutral flexion/extension alignment.

The base of the manipulandum was a Plexiglas disc encasing air jets that, when engaged, allowed frictionless wrist flexion/extension motion over the Plexiglas desktop. A single electromagnetic sensor was attached to the lateral upright of the manipulandum to measure wrist flexion/extension. The wrist joint and sensor alignments were adjusted so that the goniometric and sensor readings all indicated a neutral alignment. All electromagnetic sensors measured position with respect to a source that was mounted anterior and left of the subject on the Plexiglas desktop.

When used for wrist joint position testing, the experimental apparatus was designed to use the sensor located on the manipulandum as an indicator of wrist angle or motion.

### Testing Sequence

Subjects were tested in an "active" and a "passive" condition. Repeatability was calculated by comparing joint excursion measures during two different sessions. Testing positions included two repetitions each of ten, twenty, and thirty degrees of flexion and extension presented in a random order. In the active and passive conditions, the starting position for flexion target angles was wrist extension; and for extension target angles, the starting position was wrist flexion. For all target angles, actual location of the starting position was varied between the positioning and repositioning components of the trial to avoid subjects reproducing the extent of motion rather than joint position. Regardless of the starting position, a minimum excursion of 20° was used for all trials. All the subjects had a training session before starting the experiment in order to minimize the learning effect of the test-retest comparison.

The passive condition began when the examiner gently oscillated the subject's wrist between flexion and extension to assure relaxation. The wrist was moved to a target position and maintained for three seconds while the subject was instructed to remember the position. The wrist was once again oscillated to assure relaxation, passively placed in a different starting position, and then slowly moved toward the target position. The subject was instructed to verbally cue the examiner to stop when the wrist had reached the target position. The target position was then changed and the sequence was repeated. Wrist position was recorded at each stop.

The active condition began by placing the subject's right wrist in a starting position on the opposite side of neutral compared to the target position. The subject was asked to move the hand either "slowly toward your stomach" or "slowly away from your stomach". The subject's motion was stopped when the examiner physically restrained the manipulandum upon reaching the target position and held for three seconds while instructing the subject to remember the position. The subject was instructed not to push against the restraint. The subject was asked to relax and was passively moved to a different starting position. The subject actively returned to the target position at their desired rate of speed, indicating to the examiner when the target position was reached.

### Sensor Placement

**Figure 3:**
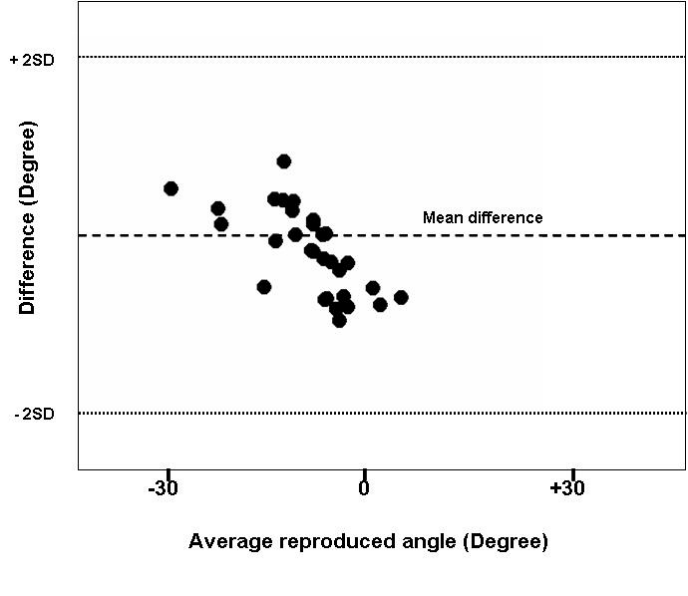
**Bland-Altman graph of manipulandum mounted sensor**. The dashed line represents the mean difference (1°) between the skin and manipulandum placement of the sensor in active condition. The dotted lines represent 2 standard deviations (SD) (+3 and -1°).

**Figure 4:**
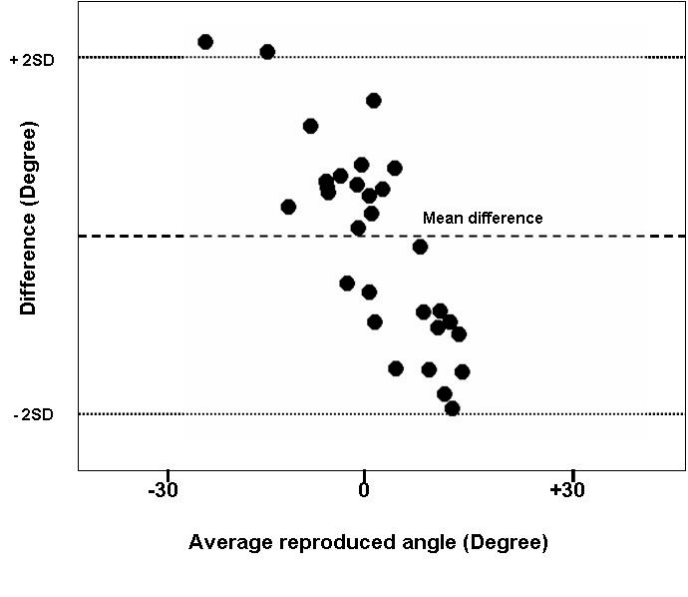
**Bland-Altman graph of skin mounted sensor**. The dashed line represents the mean difference (-2°) between the skin and manipulandum placement of the sensor in active condition. The dotted lines represent 2 standard deviations (SD) (+2 and -6°).

**Figure 5:**
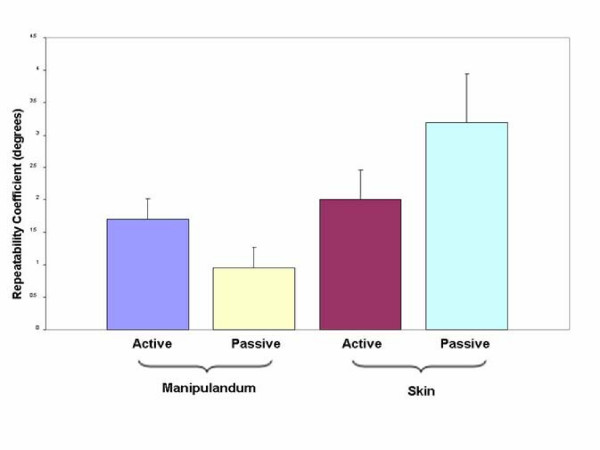
**Repeatability of range of motion measurements between sessions taking into account type of activity and sensor placements**. Repeatability coefficient is better, both in active and passive condition, when the sensors are placed on the manipulandum rather than directly on the skin.

In order to determine the best possible placement for the sensors, joint flexion/extension excursion calculated from measures taken with a manipulandum placed sensor were compared to measures from skin mounted sensors in a pilot study on five patients. A Bland-Altman Graph (a plot of the difference between reproduced angle and the mean reproduced angle) was created for both active and passive conditions to compare visually the skin and manipulandum placement (Figures 3 and 4). The purpose of Bland-Altman plots is to allow visual inspection of the data to investigate biases and to examine potential relationships between the disagreement and the true value. Then, an Intraclass Correlation Coefficient (ICC) type 2,1 with 95% confidence intervals of the test-retest repeatability between trial and between marker placement during active and passive motion and for the joint excursion measures is taken from each sensor placement during two different sessions. Measurements were captured at 30° increments through a 60° arc of motion (± 30° flexion/extension). Repeatability coefficients [39] were calculated to determine the disparity of distance moved between pairs of measures between marker sets. Arc of motion by sensors on the apparatus demonstrated greater inter-trial repeatability than did skin mounted sensors in both active and passive condition (Figure 5). We then decided to use the manipulandum mounted sensor for the rest of the experiment.

### Data Collection and Statistical Analysis

First, sample descriptive statistics (means and standard deviation) were calculated for each testing. Signed difference between the targeted position and the repositioned angle (constant error) and the absolute value of that difference (absolute error) were calculated. The standard deviation of the constant error, also known as variable error, was analyzed as an indicator of the consistency of the error. An ANOVA with repeated measure was then used to compare the results in active and passive conditions.

## Results

The average constant error in the passive condition was -0.7° ± 4.7° as compared to the active condition at 3.7° ± 5.1°. Average absolute error in the passive condition was 4.9° ± 2.9° compared to the active condition in which absolute error was 5.9° ± 3.5°. An ANOVA with repeated measures revealed significant differences between the passive and active conditions in constant (p < 0.0001) and absolute (p = 0.0084) error. Variable error in the active and passive conditions were not significantly different (passive = 4.2°; active = 4.6°; p = 0.1720). These results are summarized in Table 1.

**Table 1:** Descriptive statistics of the constant, absolute and variable error for active and passive conditions.

	Active	Passive	P value
**Constant**	3.7° ± 5.1°	-0.7° ± 4.7°	< 0.001

**Absolute**	5.9° ± 3.5°	-4.9° ± 2.9°	0.0084

**Variable**	4.6°	4.2°	0.172

## Discussion

Minimizing external influences on proprioceptive input in order to determine the effect of disease or injury has proven to be difficult. These methodological variances make it difficult to reliably isolate and quantify input from a specific structure. Clarification of the role and importance of a specific structure such as muscle spindles afferents is essential to understanding the potential impact of surgeries or injuries that diminish or destroy those structures [8,22,23].

Skin-mounted markers or electrogoniometers have been used previously in research and clinical assessment of range of motion [6,21,32-36]. These methods were deemed inappropriate for studies attempting to isolate joint contributions to proprioceptive sense because the resultant pressure and cutaneous stretch contribute redundant sensory information about limb position. Our experimental configuration minimized gravitational influences, cutaneous sensory input, and friction to emphasize, if not isolate, muscle spindles contribution to position sense in active versus passive motions. In the current experiment, subjects were not asked to precisely reproduce any joint angles. The data from this study will serve as a baseline measure of experimental setup reliability allowing future studies to differentiate subject variability over and above this demonstrated experimental variability, and to isolate joint or muscle proprioceptive inputs from each other. The technology utilized in this study (3-dimensional Flock of Birds motion tracking system) has a reported accuracy of .5° - 2° [29,30,40]. Remaining variability will be attributed to the experimental apparatus and stabilization methodology.

Errors associated with the active condition were similar to the findings of previous studies, which reported errors of 5° or less [25,27]. Values were superior to range of motion measurements obtained using an instrumented glove designed to capture hand and wrist motions which resulted in repeatability of 6.17° [41]and to goniometry which has been found to be associated with 5-8° intra- and 6-10° inter-rater reliability at the wrist [37].

Marker placement on the manipulandum appeared to result in more repeatable measures of arcs of motion than did sensors placed directly on the skin. Skin markers may have yielded variable measures because of altered sensor alignment when soft tissues were deformed during motion. For example, during wrist extension, the forearm likely pressed against the lateral support causing the skin to indent and sensor alignment to change. Also, sensor placement on the dorsal forearm could have resulted in slight sensor motion as a result of the motion or stretch on the wrist muscle tendons running under the sensor.

While it was the intent to establish concurrent validity with a tool known to provide accurate measures of motion, we propose that skin electrode placement was not the appropriate tool. It is our contention that since the use of joint repositioning to test proprioception involves comparing the difference between the angle at which the body part is placed and the angle at which it is repositioned, reliable measures are as important as precise measurement of an exact angle. Regardless of sensor placement, the passive condition was consistently associated with less precise repeatability. This methodological variability between active and passive measures forms a baseline of inaccuracy when passive limb placement is paired with active repositioning. This does not take into account the errors anticipated due to different sensory input contributing to joint position sense in active versus passive motion [21].

One explanation for the methodological differences in reproducibility associated with active and passive motions is that slight extraneous motion may have occurred at the metacarpophalangeal and interphalangeal joints during passive movement of the wrist joint via the manipulandum. In addition, the tubular dressing placed on the fingers could have stretched slightly, allowing a small amount of manipulandum rotation within the palm during passive motion. Another explanation is that the active motion, by stimulating the gamma loop, allows a more precise message encoding by the antagonist muscle spindles Ia fibers [17,42,43].

The difference in repeatability between the active and passive conditions lends support for pairing active positioning with active repositioning and passive positioning with passive repositioning when testing joint position sense. Repeatability errors of 1° in the active condition and 3° in the passive condition using manipulandum mounted markers are within acceptable ranges to allow assessment of clinically significant differences of joint position sense.

## Conclusions

The importance of proprioception in rehabilitation following musculoskeletal trauma and surgery is becoming increasingly evident, which has lead to a correspondingly increased need to understand the underlying neural mechanisms related to joint mechanics. The system utilized in the current study appears to produce an accurate and repeatable measure of active and passive motion. Differences in variability in active and passive conditions are slight with the current methodology. However, poorer reliability in passive measurements in the skin-mounted sensors lends support for the concept that active and passive motions yield different results. The primary advantage of the current system for measurement of wrist joint proprioception is that it allows the researcher to decrease extraneous influences that may affect joint position sense awareness and therefore improve the knowledge of the mechanisms underlying kinesthesia and proprioception. The results of this study indicate that the measures are repeatable and appear to be equally or more accurate than other measures previously employed to measure wrist and hand range of motion. Nevertheless, other study in order to verify the external validity of this method will be needed.

## Competing interests

The authors declare that they have no competing interests.

## Authors' contributions

AG carried out the data analysis and drafted the manuscript, KH carried out the data collection and performed the statistical analysis, KRK conceived, designed and coordinated the study, and helped with the manuscript redaction, DKH helped in the data collection and reduced the data, ERL and RAB conceived the study and participated in its design and coordination. All the authors read and approved the final manuscript.
